# Revision of the genus *Epimesoplecia* Zhang, 2007 (Diptera, Nematocera, Protopleciidae) with five new species

**DOI:** 10.3897/zookeys.492.6852

**Published:** 2015-03-30

**Authors:** Xiuqin Lin, Chungkun Shih, Dong Ren

**Affiliations:** 1 Key Lab of Insect Evolution and Environmental Change, College of Life Sciences, Capital Normal University, 105 Xisanhuanbeilu, Haidian District, Beijing 100048, China

**Keywords:** Insects, fossil, taxonomy, Jiulongshan Formation, late Middle Jurassic

## Abstract

The genus *Epimesoplecia* Zhang, 2007 of Protopleciidae is revised based on five new species, *Epimesoplecia plethora* **sp. n.**, *Epimesoplecia prosoneura* **sp. n.**, *Epimesoplecia stana* **sp. n.**, *Epimesoplecia macrostrena* **sp. n.**, and *Epimesoplecia ambloneura* **sp. n.**, described and illustrated from the Jiulongshan Formation of China. These new species, with clearly preserved characters of (1) compound eyes connected in males; (2) antennae, filiform or moniliform, with 16 segments; (3) r-m reaching the middle of the wing; (4) R_4+5_ ending very close to wing apex; (5) ratio of bRs/dRs ranging from 1.6 to 10.5; (6) M_2_ more than 3 times as long as dM_1+2_; (7) legs thin and long, femur slender, almost equal to tibia; (8) tibial spurs minute; and (9) male genitalia (previously unknown), enable us to emend the diagnosis of *Epimesoplecia* Zhang, 2007. In addition, all described species of *Epimesoplecia* are characterized, their features summarized, and a key to *Epimesoplecia* species is given.

## Introduction

Protopleciidae Rohdendorf, 1946, reported from the Jurassic, is a paraphyletic stem group to the Bibionidae (Blagoderov et al. 2002; Grimaldi and Engel 2005). Rohdendorf (1946) erected the Protopleciidae with three genera *Protoplecia* Handlirsch, 1906, *Mesoplecia* Rohdendorf, 1938 and *Mesopleciella* Rohdendorf, 1946 (Evenhuis 1994). Kovalev (1987) transferred 14 species in *Rhaetofungivora* Rohdendorf, 1964 of Pleciofungivoridae to Protopleciidae, but later, some of those were assigned to several different genera (Blagoderov 1996). The earliest record of the Protopleciidae is *Macropeza liasina* Geinitz, 1884 from the Early Jurassic in upper Liassic of Germany. Ansorge (1996) provided an updated description for *Protoplecia liasina* (Geinitz, 1884) and reported *Protoplecia klafackii* from the upper Liassic of Germany. Ansorge (1996) considered the affiliation of *Mesoplecia* and *Mesopleciella* with the Protopleciidae questionable based on the clearly shorter Sc, and suggested that *Archipleciomima* Rohdendorf, 1962 is the stem group of Pleciofungivoridae and Pleciomimidae due to long Rs stem. On the other hand, Lin (1976), Hong (1984), and Hong and Wang (1990) documented several genera and species in China, but many of them have been subsequently removed from this family (Blagoderov 1996). Recently, from the Jiulongshan Formation of China, Zhang (2007) described *Epimesoplecia* with two species, emended the diagnosis of *Mesoplecia* Rohdendorf, 1938, added two species to the genus, and excluded *Paraoligus exilus* Lin, 1976 and *Mesoplecia xinboensis* Hong, 1984 from this family, but stated that an alternative placement could not be suggested. Hao and Ren (2009) described three species of *Mesoplecia*. Lin et al. (2014) described two species as members of *Mesoplecia*, while transferring *Mesoplecia antiqua* Hao & Ren, 2009 to Mesosciophilidae, because R_2+3_ of *Mesoplecia antiqua* Hao & Ren, 2009 reaching R_1_ forming a cell r, instead of reaching the anterior margin as all other protopleciids. After documented corrections and transfers, there are 33 species in seven genera described in Protopleciidae to date (Lin et al. 2014).

Herein, based on fourteen specimens collected from the Jiulongshan Formation in Daohugou Village, Ningcheng County, Inner Mongolia, China, five new species are described in *Epimesoplecia*, *Epimesoplecia plethora* sp. n., *Epimesoplecia prosoneura* sp. n., *Epimesoplecia stana* sp. n., *Epimesoplecia macrostrena* sp. n., and *Epimesoplecia ambloneura* sp. n. with ten specimens. One of the remaining four specimens is identified as a new material for *Epimesoplecia elenae* Zhang, 2007, while the other three cannot be assigned to species owing to lack of preserved diagnostic characters. These five new species are assigned to *Epimesoplecia* by a combination of the following characters: (1) antenna long, at least twice the head length; (2) wing narrow and long; (3) Sc elongate, nearly half of wing length; (4) R_2+3_ long, more than two–thirds of R_4+5_ length.

The Jiulongshan Formation of Inner Mongolia in China is very rich in fossil insects’ record (Shi et al. 2011; Ren et al. 2010; Liu et al. 2012; Wang et al. 2013 and Li et al. 2013). Because of new calibrations for the Jurassic System, this deposit should be now considered as latest Middle Jurassic (late Callovian) in age (Walker et al. 2013). The paleoenvironment reconstructed for that time was a volcanic region with mountains, streams and lakes under a humid and warm climate (Ren et al. 2002; Gao and Ren 2006).

## Material and methods

**Figure 1.**
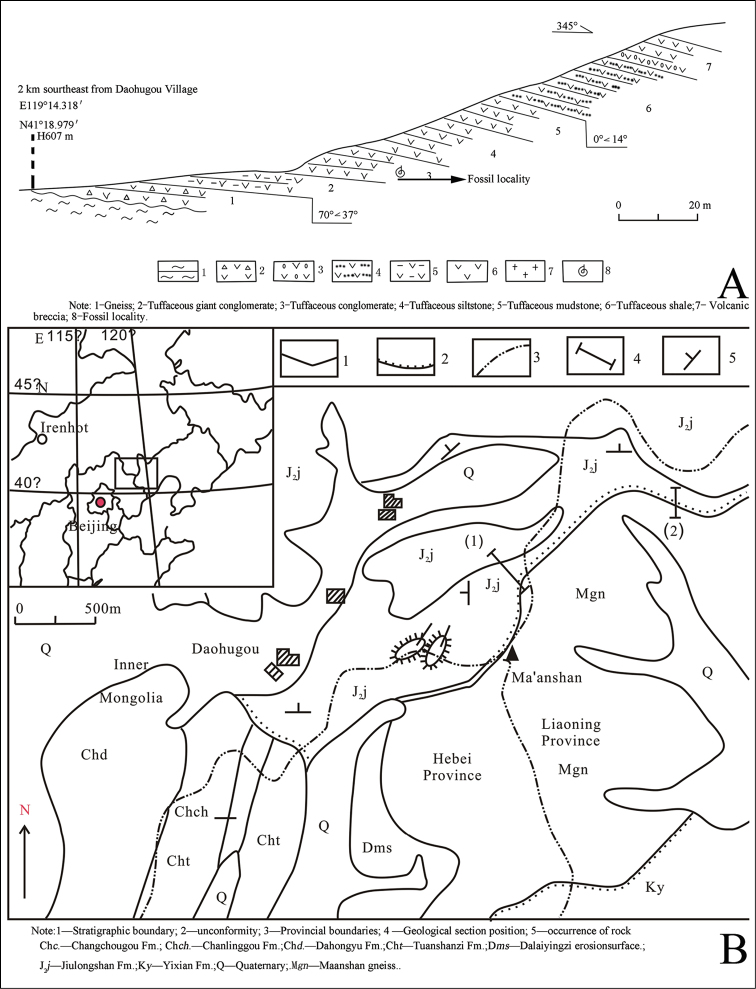
**A** Measured stratigraphic section at the Jiulongshan Formation of northeastern China **B** Map showing the fossil locality (after Ren et al. 2002).

All the type materials were collected from the Jiulongshan Formation (Fig. 1A) of Daohugou Village in Ningcheng County of Inner Mongolia, China (Fig. 1B) (after Ren et al. 2002). The specimens are housed in the Key Laboratory of Insect Evolution and Environmental Changes, College of Life Sciences, Capital Normal University, Beijing, China (CNUB; Dong Ren, Curator). The specimens were examined and photographed using a Leica MZ12.5 dissecting microscope with a Leica DFC 500 digital camera and illustrated with the aid of camera lucida attached to the microscope. The line drawings were drawn by Adobe Photoshop CS5. The wing venation nomenclature used in this paper is based on the interpretations and system proposed by Shcherbakov et al. (1995) and Wootton and Ennos (1989).

## Systematic Paleontology

### Order Diptera Linnaeus, 1758 Suborder Nematocera Latreille, 1825 Family Protopleciidae Rohdendorf, 1946

#### Genus Epimesoplecia

Taxon classificationAnimaliaDipteraProtopleciidae

Zhang, 2007

##### Type species.

*Epimesoplecia shcherbakovi* Zhang, 2007

##### Species included.

Type species, *Epimesoplecia elenae* Zhang, 2007, *Epimesoplecia plethora* sp. n., *Epimesoplecia prosoneura* sp. n., *Epimesoplecia stana* sp. n., *Epimesoplecia macrostrena* sp. n. and *Epimesoplecia ambloneura* sp. n.

##### Revised diagnosis.

Compound eyes connected in males. Antennae filiform or moniliform, with 16 segments, at least twice of head length or slightly less than twice of head length; wings narrow and long; Sc elongate, at or near the same level of r-m; bRs at least 4 times as long as r-m; R_2+3_ long, more than two-thirds of R_4+5_, R_2+3_ slightly sigmoidly curved or straight, reaching anterior margin distad of the apex of R_1_; r-m reaching the middle of the wing; R_4+5_ ending very close to wing apex; M_1+2_ furcated distinctly proximad or distad of R_2+3_; M_2_ more than 3 times as long as dM_1+2_; bM_3+4_ longer or slightly shorter than m-cu; pterostigma absent; bM_1+2_ longer or shorter than dM_1+2_. Legs thin and long, femur slender, almost equal to tibia; tibial spurs minute. Male genitalia: abdomen cylindrical; genitalia complex, narrower than the 8th segment, with gonocoxites rounded; gonostylus elongated, shorter than gonocoxites. Female genitalia: the 8th segment smaller than preceding segments, genitalia simple, with 2-segmented cerci, the basal segment of cerci longer than the terminal one.

#### Epimesoplecia plethora sp. n.

Taxon classificationAnimaliaDipteraProtopleciidae

http://zoobank.org/86178CAD-809C-4637-B64A-33B6BFBA6030

Figs 2 , 3

##### Etymology.

The epithet of *plethora* is derived from the Greek word “plethore”, meaning “fullness”, emphasizing the body covered with dense pubescence. The specific epithet is a noun in apposition.

##### Diagnosis.

Compound eyes crescent. Antennae moniliform. Sc very close to the level of r-m; fork of Rs distad of fork of M_1+2_; Rs distad of crossvein r-m; bRs less than 2 times (1.6–1.8) as long as dRs, the latter about 3 times (2.6–3) as long as r-m; R_2+3_, sigmoidly curved, distinctly shorter than bRs and dRs combined; bM_1+2_ shorter than dM_1+2_; bM_3+4_ shorter than m-cu; cell bp as wide as cell ba terminally.

##### Material.

**Figure 2.**
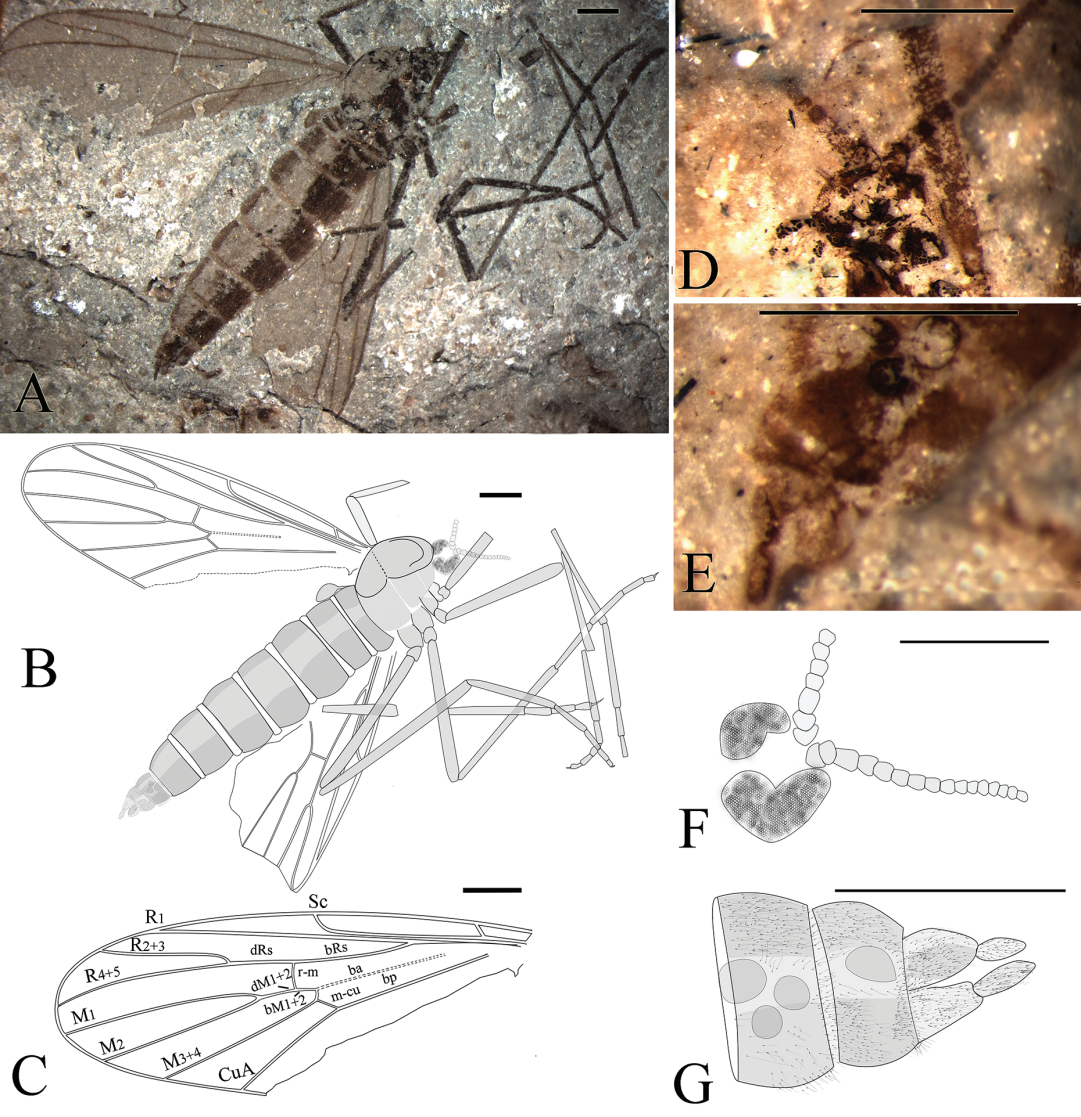
*Epimesoplecia plethora* sp. n. (CNU-DIP-NN2013202). Holotype. **A** Photograph of habitus; Line drawings of **B** Habitus **C** Left wing; Photographs of **D** Details of head (under alcohol) **E** Details of female genitalia (under alcohol); Line drawings of **F** Head **G** Female genitalia. Scale bars = 1 mm.

**Figure 3.**
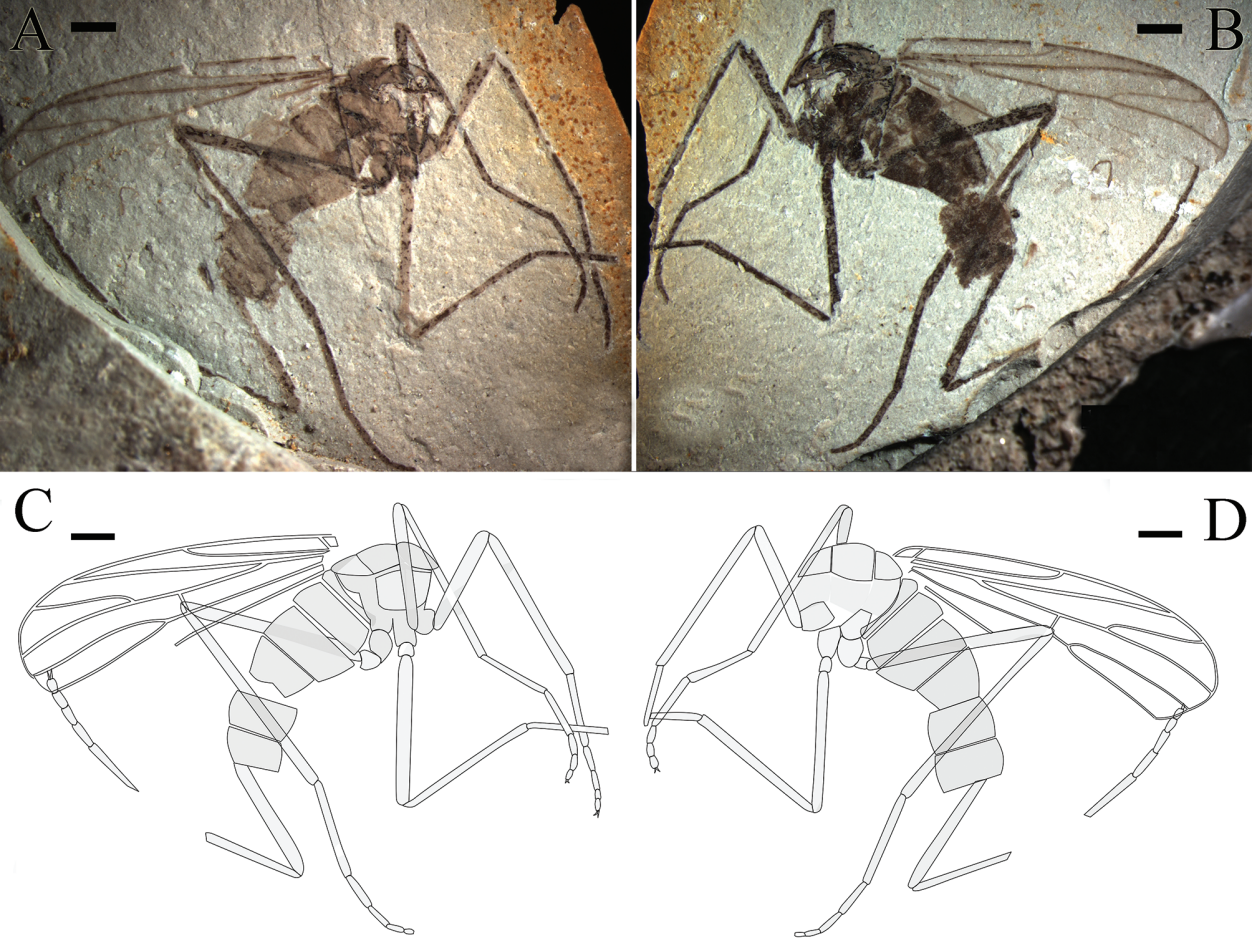
*Epimesoplecia plethora* sp. n. (CNU-DIP-NN2013209p/c). Paratype. **A, B** Photographs of part and counterpart **C, D** Line drawings of part and counterpart. Scale bars = 1 mm.

Holotype: Female. NO. CNU-DIP-NN2013202, a well-preserved almost complete body with left haltere, both wings and part of legs (Fig. 2A). Paratype: NO. CNU-DIP-NN2013209p/c, part and counterpart, lateral view, only right wing and legs preserved, head and abdomen incomplete (Fig. 3A, B).

##### Horizon and locality.

All specimens were collected from the Jiulongshan Formation, late Middle Jurassic age (Late Callovian) from Daohugou Village, Ningcheng County, Inner Mongolia Autonomous Region in China.

##### Description.

**Head** (Fig. 2D, F): Oviform. Compound eyes crescent in females. Antennae: scape and pedicel thick and stout, 1st flagellomere slender, the remaining ones becoming thinner toward apex.

**Thorax** (Figs 2A, 3A, B): Scutum convex; scutellum clearly projecting; haltere depressed.

**Legs** (Figs 2A, 3A, B): Forelegs comparatively thin and slender, femur slightly thicker than tibia, covered with dense setae as preserved. The 1st tarsomere 2 times as long as the 2nd tarsomere; the 3rd to 5th tarsi gradually thinned, claws small. Mid legs: femur long and slender, almost equal to tibia, tibial spurs minute, claws well-preserved. Hind legs: femur more than four-fifths of tibia; distinctly longer than forelegs and mid legs, tibia less than 2 times as long as femur; the 1st tarsomere more than 2 times as long as the 2nd tarsomere; with two pretarsal claws.

**Wings** (Figs 2A, C and 3A): Wing long and narrow, 2.6–2.8 times as long as width (length 8.4–9.0 mm, width 3.0–3.4 mm); Sc terminating at the middle of the anterior margin, the costal field narrow; bRs 4–5 times as long as r-m; R_2+3_ slightly sigmoidly curved; Rs arising from one-fourth of wing length, furcating distal level of fork of M_1+2_; stem of Rs longer than stem R, the former longer than R_2+3_; R_4+5_ weakly curved upward medially, ending just below apex of wing; both R_4+5_ and M_1_ subparallel; crossvein m-cu as long as r-m; CuA strongly curved, distad of M forking, ending at posterior margin of wing; A_1_ not preserved.

**Female genitalia** (Fig. 2E, G): The 8th segment slightly smaller than preceding segments, genitalia simple, with 2-segmented cerci, the basal segment of cerci thicker and longer than the terminal one.

##### Dimensions

**(in mm).** [Measurements for the paratype CNU-DIP-NN2013209p/c in brackets, if different]. Holotype: female. CNU-DIP-NN2013202, Body length 10 [5 as preserved], maximal width of body 2.2 [2.4]. Head length 0.6, width 0.8. Forelegs: femur 1.7 as preserved [2.2]; tibia 1.4 as preserved [3.4]. Mid legs: femur 3 [2.8], tibia 3.5 [3]. Hind legs: femur 3.6 [4], tibia 4.4 [5]. Wing: length 9.0 [8.2], width 3.4 [3.0], R_2+3_ 2.4, bRs 2.2 [2.1], dRs 1.2, R_4+5_ 3 [3.4].

##### Remarks.

The new species is differentiated from *Epimesoplecia shcherbakovi* Zhang, 2007 by the following features: bRs less than 2 times (1.6–1.7) as long as dRs (vs. bRs 4.5 times as long as dRs); Rs bifurcation distad to fork of M_1+2_ (vs. Rs bifurcation at the same level of fork of M_1+2_); dM_1+2_ longer than bM_1+2_ (vs. dM_1+2_ shorter than bM_1+2_). The new species differs from *Epimesoplecia elenae* Zhang, 2007 in having antennae moniliform (vs. filiform); bRs short, less than 2 times (1.6–1.7) as long as dRs (vs. bRs long, 2.5 times as long as dRs); bM_3+4_ clearly shorter than m-cu (vs. bM_3+4_ as long as m-cu). Comparisons with other species are listed in Table 1.

**Table 1.** Summary of data for all species of *Epimesoplecia* Zhang, 2007.

Species	Specimen numbers	H/P	Sex	BL	WL	L/W	bRs/dRs	dRs/r-m	bM_1+2_/dM_1+2_	M_2_/dM_1+2_	bM_3+4_/m-cu	bRs/r-m	Rs vs. M_1+2_
*Epimesoplecia plethora* sp. n.	CNU-DIP-NN2013202	H	♀	10.6	9	2.6	1.6	2.6	0.6	4.2	shorter	4	DS
	CNU-DIP-NN2013209 p/c	P	NA	5 (ic)	8.4	2.8	1.75	3	0.8	6	NA	5	DS
*Epimesoplecia prosoneura* sp. n.	CNU-DIP-NN2013207 p/c	H	♂	8.3	8	3.1	10.5	0.3	0.4	4.2	shorter	4.8	PX
	CNU-DIP-NN2013214	A	♀	8.2	8.9	3.3	9.4	0.6	0.8	5.2	shorter	5.8	PX
*Epimesoplecia stana* sp. n.	CNU-DIP-NN2013201 p/c	H	♀	10.2	11.2	3.3	2.5	2	2.3	8	shorter	5.4	DS
*Epimesoplecia shcherbakovi* Zhang, 2007	DHG200384	H	NA	7 (ic)	9.7	2.7	4.5	0.9	1.5	5	longer	4.6	SL
*Epimesoplecia elenae* Zhang, 2007	DHG200385	H	♀	10.5	10	2.4	2.5	1.8	0.6	5	shorter	4	DS
	CNU-DIP-NN2013213 p/c	N	♀	11.2	7.6	2.5	2.7	1.7	0.5	5.6	shorter	4.6	DS
*Epimesoplecia macrostrena* sp. n.	CNU-DIP-NN2013211	H	NA	9(ic)	7.4(ic)	>3	5	1.1	0.7	≈3	NA	5.6	PX
	CNU-DIP-NN2013212	P	♀	10.3	7.1	2.5	4.7	1.1	0.7	3	shorter	4.6	PX
	CNU-DIP-NN2013206 p/c	P	NA	7 (ic)	8	3.5	4.2	1.2	0.7	6.2	longer	4.4	PX
*Epimesoplecia ambloneura* sp. n.	CNU-DIP-NN2013215	H	♀	12.7	9.1	2.5	7	0.7	2.8	11	shorter	5	SL
	CNU-DIP-NN2013208	P	NA	5 (ic)	7.4	2.3	6	0.7	1.3	8	shorter	5	SL

Notes: Abbreviations: 1. H = Holotype, P = Paratype, A = Allotype, N = New material; 2. BL = Body length (mm); 3. WL = Wing length (mm); 4. L/W = The ratio of wing length and width; 5. ic = incomplete; 6. p/c = Part and counterpart; 7. DS = Fork of Rs distad of fork of M_1+2_; 8. PX = Fork of Rs proximad of fork of M_1+2_; 9. NA = Not Available; 10. SL = Rs and M_1+2_ at the same level.

#### Epimesoplecia prosoneura sp. n.

Taxon classificationAnimaliaDipteraProtopleciidae

http://zoobank.org/260A2E9B-8331-483A-9BBB-E67D58EB0B32

Figs 4 , 5

##### Etymology.

The epithet of *prosoneura* is derived from the Greek preposition “pro”, meaning “before”, and Greek word “neura”, meaning “string or sinew”, referring to proximal position of the fork of Rs. The specific epithet is a noun in apposition.

##### Diagnosis.

Compound eyes crescent. Antennae moniliform. Sc exceeding the level of r-m or very close to r-m; fork of Rs proximad of fork of M_1+2_; R_2+3_ very close to crossvein r-m; R_2+3_, almost straight, distinctly longer than bRs and dRs combined; bRs about 10 times (9.4–10.5) as long as dRs, the latter as long as r-m; bM_1+2_ shorter than dM_1+2_; bM_3+4_ shorter than m-cu; cell bp wider than cell ba terminally.

##### Material.

**Figure 4.**
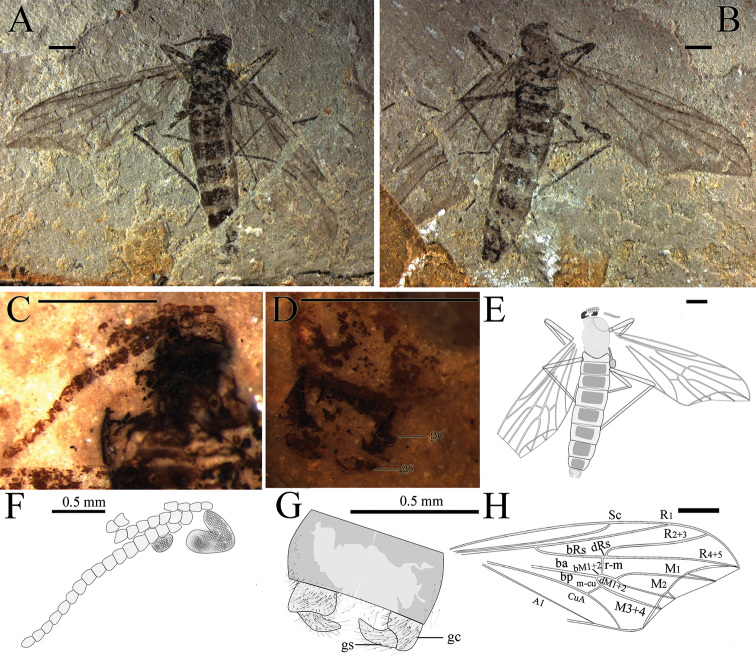
*Epimesoplecia prosoneura* sp. n. (CNU-DIP-NN2013207p/c). Holotype. Photographs of **A, B** Part and counterpart **C** Details of head (under alcohol) **D** Details of male genitalia (under alcohol); Line drawings of **E** Counterpart **F** Head **G** Male genitalia **H** Wing. Scale bars = 1 mm. gc–gonocoxite; gs–gonostylus.

**Figure 5.**
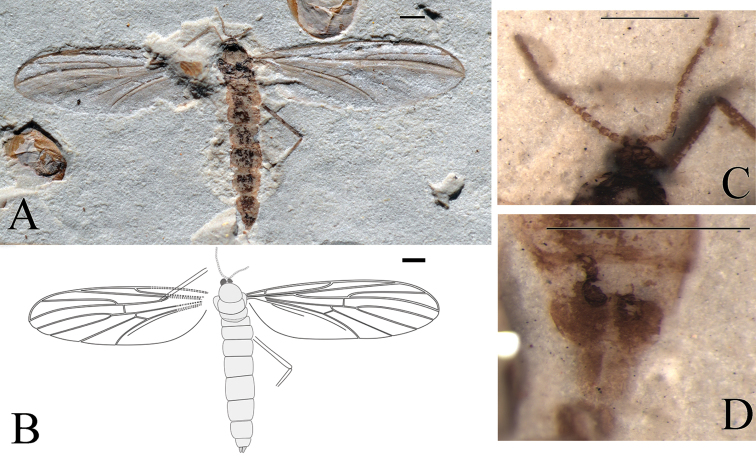
*Epimesoplecia prosoneura* sp. n. (CNU-DIP-NN2013214). Allotype. **A** Photograph of habitus **B** Line drawing of habitus; Photographs of **C** Details of head (under alcohol); **D** Details of female genitalia (under alcohol). Scale bars = 1 mm.

Holotype: male, NO. CNU-DIP-NN2013207p/c, part and counterpart, an almost complete specimen with well-preserved antennae, both wings and part of legs (Fig. 4A, B). Allotype (paratype): female. NO. CNU-DIP-NN2013214, in dorsal view, a specimen with well-preserved wings and body (Fig. 5A, B).

##### Horizon and locality.

All specimens were collected from the Jiulongshan Formation, late Middle Jurassic age (Late Callovian) from Daohugou Village, Ningcheng County, Inner Mongolia Autonomous Region in China.

##### Description.

**Head** (Figs 4C and 5C): Oviform. Compound eyes protrusive in males. Antennae long, scape and pedicel thick and stout, the 1st flagellomere slender and long, the remaining ones becoming thinner toward apex.

**Thorax** (Figs 4A, B and 5A): Prothorax barely visible; scutum of mesothorax broad and oval, convex obviously; scutellum of metathorax projecting, semicircle; haltere depressed.

**Legs** (Figs 4A, B and 5A): Forelegs relatively slender; femur slender and long, covered with dense setae, slightly shorter than tibia; tarsi not preserved. Mid legs similar to forelegs, femur long and slender, tibia thinner than femur as preserved; Hind legs: femur slightly expanded, tibia slightly longer than femur as preserved.

**Wings** (Figs 4H and 5B): Wing long and narrow (length: 8–8.9 mm, width: 2.6–2.7 mm), apex of wings covering the abdominal terminalia. Costal field long and thin, Sc reaching C at the middle of anterior margin; Rs arising from basal one-fourth of wing length, furcating distad to fork of M_1+2_; bRs about 5 times (4.8–5.7) as long as r-m; crossvein m-cu slightly longer than r-m; CuA slightly curved, ending at posterior margin distad of mid wing; vein A_1_ nearly straight, reaching posterior margin.

**Male genitalia** (Fig. 4G): Abdomen cylindrical; genitalia complex, slightly narrower than the 8th segment, with gonocoxites robust and rounded; gonostylus cylindrical and elongated, shorter than gonocoxites.

**Female genitalia** (Fig. 5D): The 8th segment slightly smaller than preceding segments, genitalia simple, with 2-segmented cerci, the basal segment of cerci longer than the terminal one.

##### Dimensions of holotype

**(in mm).** [Measurements for the paratype CNU-DIP-NN2013214 in brackets, if different]. Holotype: male, CNU-DIP-NN2013207p/c, Body length 9.3 [8.2], maximal width of body 1.6 [1.4]. Antennae length: 1.8 (segments 1–16) [1.4 (segments 1–15)]. Foreleg: femur 1.6 as preserved; tibia 2.5 as preserved. Mid leg: femur 1.9 as preserved; tibia 2.7 as preserved. Hind leg: femur 2.2 as preserved [2 as preserved], tibia 3.5 as preserved [1.3 as preserved]. Wing: length 8 [8.9], width 2.6 [2.7]; R_2+3_ 3 [3.7]; bRs 1.9 [2.3]; dRs 0.1 [0.3]; R_4+5_ 3.6 [4].

##### Remarks.

The new species is similar to *Epimesoplecia shcherbakovi* Zhang, 2007 but differs from the latter in having bRs about 10 times (9.4–10.5) as long as dRs (vs. 4.5 times); Rs bifurcation proximad of fork of M_1+2_ (vs. Rs bifurcation at the same level of fork of M_1+2_); dM_1+2_ longer than bM_1+2_ (vs. dM_1+2_ shorter than bM_1+2_); R_2+3_, very close to the position crossvein r-m, distinctly longer than bRs and dRs combined (vs. R_2+3_, distad of the position crossvein r-m, clearly shorter than bRs and dRs combined). The new species differs from *Epimesoplecia plethora* sp. n. in having bRs about 10 times (9.4–10.5) as long as dRs (vs. less than 2 times); bM_3+4_ clearly shorter than m-cu (vs. bM_3+4_ shorter than m-cu); Rs bifurcation proximad of fork of M_1+2_ (vs. Rs bifurcation distad of fork of M_1+2_); dRs distinctly shorter than r-m (vs. dRs about 3 times as long as r-m); R_2+3_, very close to the position crossvein r-m, distinctly longer than bRs and dRs combined (vs. R_2+3_, sigmoidly curved, distinctly shorter than bRs and dRs combined). Comparisons with other species are listed in Table 1.

#### Epimesoplecia stana sp. n.

Taxon classificationAnimaliaDipteraProtopleciidae

http://zoobank.org/323B8EC8-B13C-4360-BCFF-82E436529BB0

Fig. 6

##### Etymology.

The epithet is an arbitrary combination of letters used as a nun in apposition.

##### Diagnosis.

Antennae moniliform. Sc very close to the level of r-m; fork of Rs distad of fork of M_1+2_; R_2+3_ distad of crossvein r-m; R_2+3_, straight, distinctly less than bRs and dRs combined; bRs 2.5 times as long as dRs, the latter 2 times as long as r-m; bM_1+2_ significantly longer than dM_1+2_ (2.3 times); bM_3+4_ slightly longer than m-cu; cell bp narrower than cell ba terminally.

##### Material.

Holotype: female, NO. CNU-DIP-NN-2013201p/c, in lateral view, a well-preserved body with partial antennae, almost complete wings and legs.

##### Horizon and locality.

The specimen was collected from the Jiulongshan Formation, late Middle Jurassic age (Late Callovian) from Daohugou Village, Ningcheng County, Inner Mongolia Autonomous Region in China.

##### Description.

**Figure 6.**
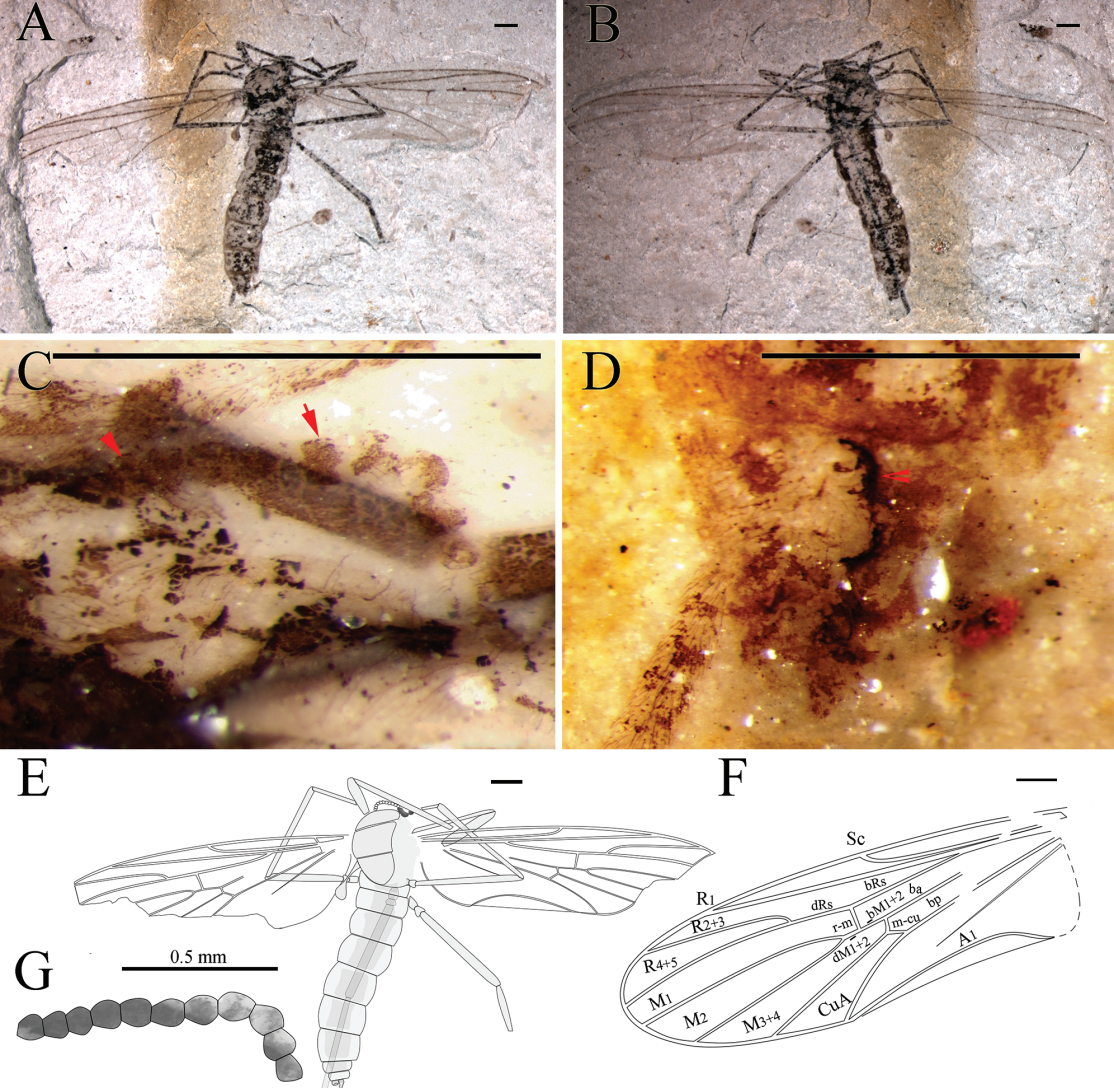
*Epimesoplecia stana* sp. n. (CNU-DIP-NN2013201p/c). Holotype. Photographs of **A, B** Part and counterpart; **C** Details of head (under alcohol) **D** Details of female genitalia (under alcohol); Line drawings of **E** Part **F** Left wing of counterpart **G** Partial antenna. Scale bars = 1 mm.

**Head** (Fig. 6C): small, in lateral view; Antennae with segments 1–11 visible as preserved, moniliform. Maxillary palpi barely visible.

**Thorax** (Fig. 6A, B): Prothorax barely visible; scutum of mesothorax broad and oval, convex obviously; scutellum of metathorax projecting, semicircular; haltere depressed.

**Legs** (Fig. 6A, B): Forelegs relatively slender, femur slightly expanded, slightly shorter than tibia; tarsi not preserved. Mid legs similar to forelegs, femur thicker than tibia; tibial spurs minute; tarsi not preserved. Hind legs: femur slightly expanded, slightly shorter than tibia; tibia slender and long as preserved.

**Wings** (Fig. 6F): Wing long and narrow, more than 3 times as long as width (length: 11.2 mm, width: 3.4 mm as preserved); apex of wings covering the abdominal terminalia. Costal field long and thin, Sc reaching C very close to the position of r-m; Rs arising from basal one-fourth of wing length, furcating distad to level of fork of M_1+2_. Stem of Rs longer than stem of R, the former longer than R_2+3_, bRs 5 times as long as r-m; crossvein r-m longer than m-cu; CuA slightly curved, ending at posterior margin; vein A_1_ short, slightly longer than half of cell bp.

**Female genitalia** (Fig. 6D): The 8th segment slightly smaller than preceding segments, genitalia simple, cerci segments not visible.

##### Dimensions

**(in mm).** Holotype: female, CNU-DIP-NN2013201p/c, Body length 10.2, maximal width of body 2.2. Antennae 1.1 (segments 1–11). Foreleg: femur 2.5; tibia 2.7. Mid leg: femur 3; tibia 3.4. Hind leg: femur 3.7, tibia 3.4. Wing: length 11.2, width 3.4; R_2+3_ 3; bRs 2.5; dRs 1.1; R_4+5_ 3.7.

##### Remarks.

The new species having ratio of bRs/dRs of 2.5 is similar to *Epimesoplecia elenae* Zhang, 2007, but is distinguished from the latter by having Rs bifurcation significantly distad of fork of M_1+2_ (vs. Rs bifurcation slightly distad of fork of M_1+2_); bM_1+2_ distinctly longer than dM_1+2_ (vs. bM_1+2_ clearly shorter than dM_1+2_); dM_1+2_ clearly shorter than r-m (vs. dM_1+2_ as long as r-m); dM_1+2_ short, almost one-eighth of M_2_ (vs. dM_1+2_ long, one-fifth of M_2_). Comparisons with other species are listed in Table 1.

#### Epimesoplecia macrostrena sp. n.

Taxon classificationAnimaliaDipteraProtopleciidae

http://zoobank.org/8A937A8A-0349-45A6-B5FB-D5287856A37F

Figs 7 , 8

##### Etymology.

The epithet of *macrostrena* is derived from the Greek prefix “macro-”, meaning “large”, and Greek word “strenos”, meaning “insolence or excess of strength”, referring to the large wings and strong body of this species. The specific epithet is a noun in apposition.

##### Diagnosis.

Antennae filiform. Sc very close to the level of r-m; fork of Rs proximad of fork of M_1+2_; R_2+3_ distad of crossvein r-m; R_2+3_, straight, slightly shorter than bRs and dRs combined; bRs about 5 times (4.2–5) as long as dRs, the latter almost equal to r-m; bM_1+2_ shorter than dM_1+2_ (0.7 times); bM_3+4_ shorter than m-cu (barely longer than m-cu); cell bp slightly wider than cell ba terminally.

##### Materials.

**Figure 7.**
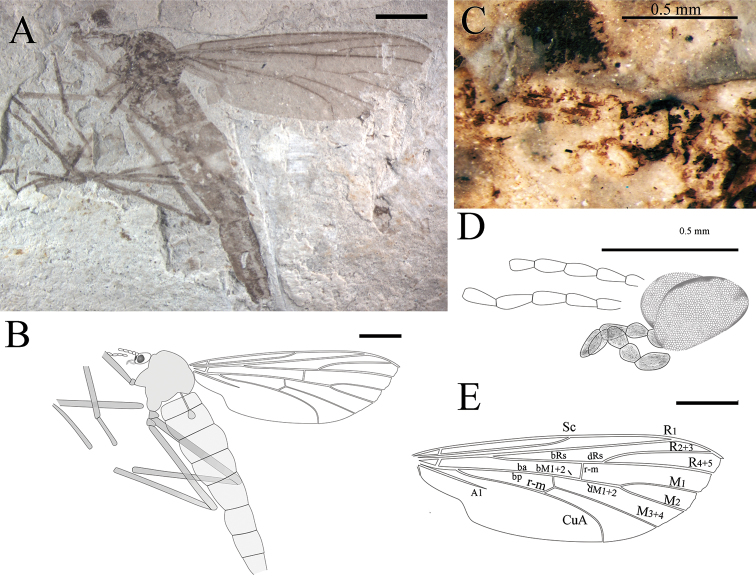
*Epimesoplecia macrostrena* sp. n. (CNU-DIP-NN2013211). Holotype. **A** Photograph of habitus **B** Line drawing of habitus **C** Photograph of details of head (under alcohol); Line drawings of **D** Head **E** Wing. Scale bars = 1 mm.

**Figure 8.**
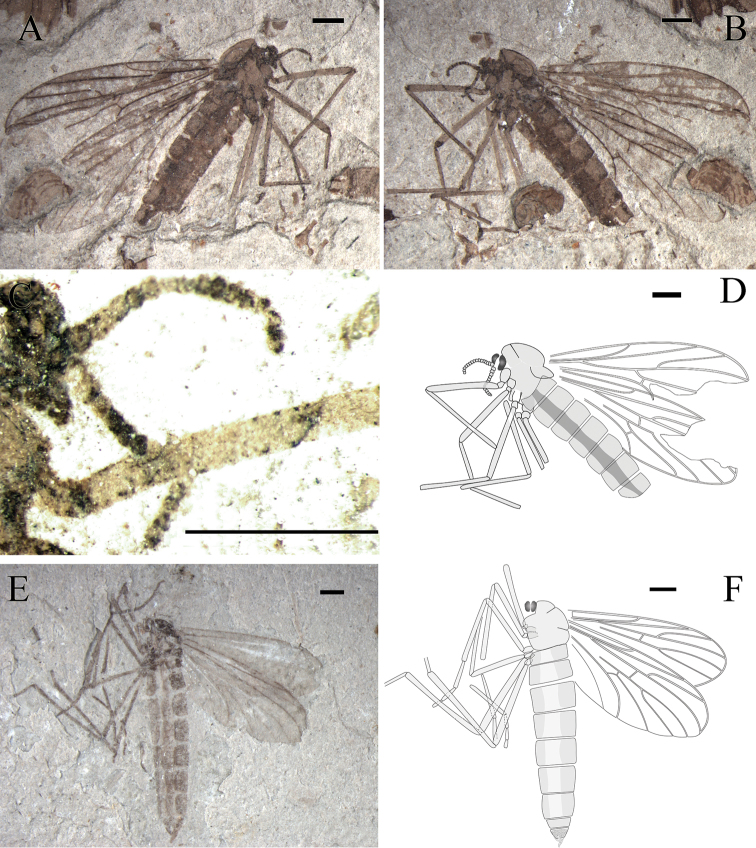
*Epimesoplecia macrostrena* sp. n. (CNU-DIP-NN2013206p/c). Paratype. Photographs of **A, B** Part and counterpart **C** Details of head; Line drawing of **D** Counterpart; (CNU-DIP-NN2013212) Paratype **E** Photograph of habitus **F** Line drawing of habitus. Scale bars = 1 mm.

Holotype: sex unknown. NO. CNU-DIP-NN-2013211, in lateral view, a well-preserved specimen with partial antennae, wings and body as preserved (Fig. 7). Paratypes: CNU-DIP-NN-2013206p/c, sex unknown, a well-preserved specimen with almost complete wings and body (Fig. 8A, B), NO. CNU-DIP-NN-2013212, female, in ventral view, a well-preserved specimen with complete wings and body as preserved (Fig. 8E).

##### Horizon and locality.

All specimens were collected from the Jiulongshan Formation, late Middle Jurassic age (Late Callovian) from Daohugou Village, Ningcheng County, Inner Mongolia Autonomous Region in China.

##### Description.

**Head** (Figs 7C, D and 8C): Oviform and very small in lateral view; antennae filiform, segments 1–9 well-preserved, scape and pedicel slightly compressed; flagellar segments slender and long, becoming thinner and shorter toward apex (moniliform in CNU-DIP-NN-2013206p/c in Fig. 8C); maxillary palpi segments not visible.

**Thorax** (Figs 7A, B and 8E): Scutum well-developed, arched convex; scutellum depressed; haltere clearly depressed.

**Legs** (Figs 7A, B and 8E): Forelegs: femur thicker than tibia; almost four-fifths of tibia; the 1st tarsomere longer than half of tibia, the 2nd–5th greatly thinned than the former. Mid legs: femur slightly thicker than tibia; tibial spurs minute; the 1st tarsomere slightly longer than the 1st tarsomere of forelegs. Hind legs: femur expanded almost equal to tibia; tibial spurs minute; tarsi not preserved.

**Wings** (Figs 7E, 8D and F): Wing long and narrow (length: 7.1–8 mm, width: 2.3–3.2 mm), apex of wings not reaching the abdominal terminalia. Costal field long and thin, apex of Sc gradually tapering to the end; Rs arising almost from basal one-fifth of wing length, furcating proximad of fork of M_1+2_ bRs 4.4–5.6 times as long as r-m; crossvein m-cu slightly shorter than r-m; CuA arched near anal margin; vein A_1_ nearly straight, reaching posterior margin.

**Female genitalia** (Fig. 8F): In lateral view, genitalia simple, with 2-segmented cerci.

##### Dimensions

**(in mm).** [Measurements for the holotype in brackets]. Body length 7 (as preserved)–11.3 [10.3 as preserved]; antennae 0.9 (as preserved)–1.4 (segments 1–16) [0.9 segments 1–9]. Forelegs: femur 2.1–2.5 [1.8 as preserved]; tibia 2.9–3.1 [2.7 as preserved]. Mid leg: femur 2.4–3.8 as preserved [3.8 as preserved]; tibia 2.7–3.6 as preserved [3.6 as preserved]. Hind leg: femur 3.4–4.7 [4.7], tibia 3.8–4.8 [4.8]. Wing: length 7.1–8 [7.4 as preserved], width 2.3–3.3 [3.3]; R_2+3_ 2.4–3.2 [3.2]; bRs 1.9–2.9 [2.9]; dRs 0.5–0.8 [0.6]; R_4+5_ 3–3.7 [3.7].

##### Remarks.

The new species is similar to *Epimesoplecia shcherbakovi* Zhang, 2007, but is differentiated from the latter by having Rs bifurcation proximad of fork of M_1+2_ (vs. Rs bifurcation at the same level of fork of M_1+2_); bM_1+2_ shorter than dM_1+2_ (vs. bM_1+2_ longer than dM_1+2_); dRs as long as r-m (vs. dRs clearly shorter than r-m); dM_1+2_ long, almost one-third of M_2_ (vs. dM_1+2_ short, significantly less than one-third of M_2_). Comparisons with other species are listed in Table 1.

#### Epimesoplecia ambloneura sp. n.

Taxon classificationAnimaliaDipteraProtopleciidae

http://zoobank.org/268EE32F-E347-45C1-8FF7-F3DC03805D09

Figs 9 , 10

##### Etymology.

The epithet of *ambloneura* is derived from the Greek prefix “ambl-”, meaning “obtuse”, and Greek word “neura”, meaning “string or sinew”, referring to the blunt caudal vein of this species. The specific epithet is a noun in apposition.

##### Diagnosis.

Antennae filiform. Sc very close to the level of r-m; fork of Rs at the same level of fork of M_1+2_; R_2+3_ proximad of crossvein r-m; R_2+3_, straight, distinctly longer than bRs and dRs combined; bRs about 6–7 times as long as dRs, the latter clearly shorter than r-m; bM_1+2_ significantly longer than dM_1+2_ (1.3–2.8 times); M_2_ 8–11 times as long as dM_1+2_; bM_3+4_ shorter than m-cu; cell bp slightly wider than cell ba terminally.

##### Materials.

**Figure 9.**
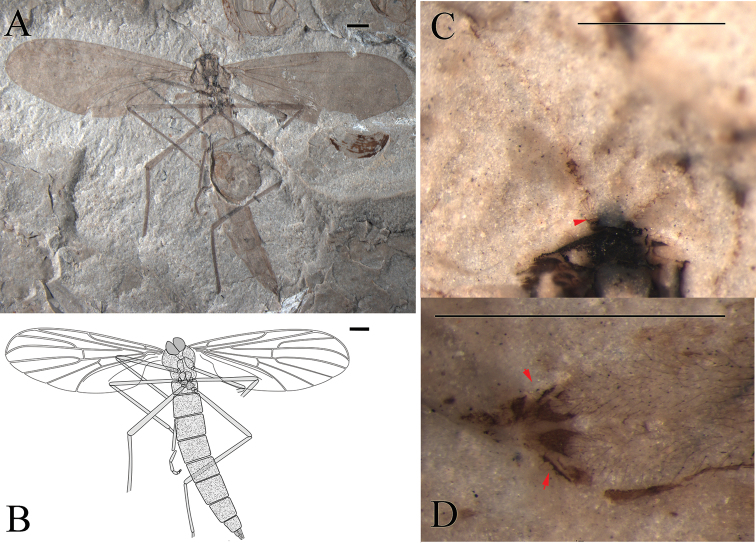
*Epimesoplecia ambloneura* sp. n. (CNU-DIP-NN2013215). Holotype. **A** Photograph of habitus **B** Line drawing of habitus; Photographs of **C** Details of head (under alcohol) **D** Details of female genitalia (under alcohol). Scale bars = 1 mm.

**Figure 10.**
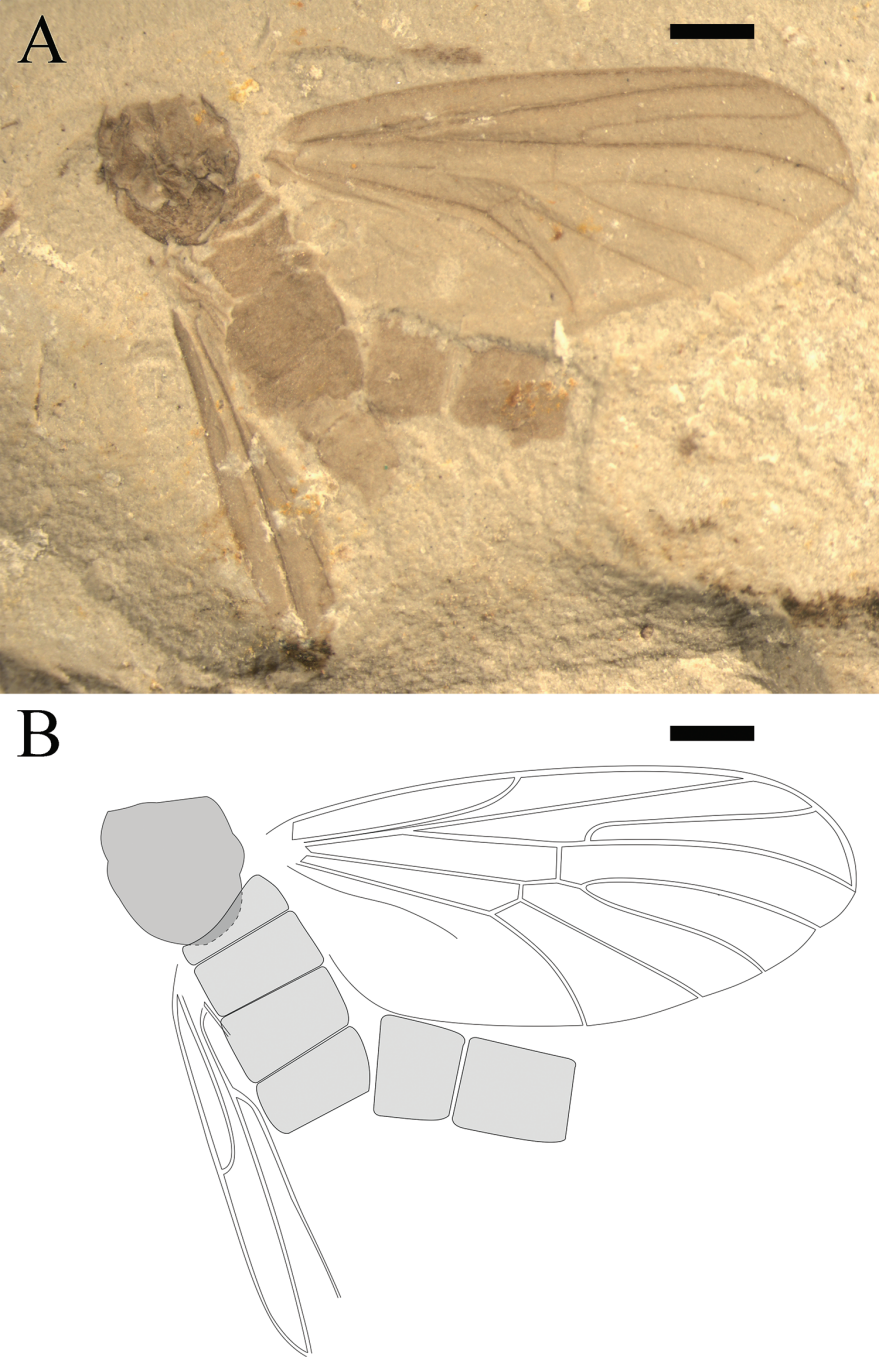
*Epimesoplecia ambloneura* sp. n. (CNU-DIP-NN2013208). Paratype. **A** Photograph of habitus **B** Line drawing of habitus. Scale bars = 1 mm.

Holotype: male. NO. CNU-DIP-NN-2013215, in ventral view, a well-preserved specimen with partial antennae, complete wings and body (Fig. 9A). Paratype: sex unknown. NO. CNU-DIP-NN-2013208, in dorsal view, a specimen with only right wing well-preserved, but fragments of body as preserved (Fig. 10).

##### Horizon and locality.

All specimens were collected from the Jiulongshan Formation, late Middle Jurassic age (Late Callovian) from Daohugou Village, Ningcheng County, Inner Mongolia Autonomous Region in China.

##### Description.

**Head** (Fig. 9C): Oviform and very small in lateral view; antennae long, with 16 segments, scape and pedicel slightly compressed; flagellar segments slender and long, becoming thinner and shorter toward apex; maxillary palpi segments barely visible.

**Thorax** (Figs 9A and 10A): Scutum well-developed, arched convex; scutellum depressed; haltere not visible.

**Legs** (Fig. 9A): Forelegs: femur slightly thicker than tibia; almost equal to tibia; tarsi not preserved. Mid legs: femur thicker than tibia; tibial spurs minute; tarsi not preserved. Hind legs: femur expanded, almost equal to tibia; tibial spurs minute; the 1st tarsomere longer than half of tibia; tarsi 2nd–5th not preserved.

**Wings** (Figs 9B and 10B): Wing long and narrow (length: 7.4–9.1 mm, width: 3.2–3.6 mm), apex of wings not reaching the abdominal terminalia. Costal field long and thin, apex of Sc gradually tapering to the end; Rs arising almost from basal one-fifth of wing length, furcating at the same level of fork of M_1+2_; bRs 5 times as long as r-m; CuA slightly oblique; vein A_1_ nearly straight, not reaching posterior margin, exceeding the level of m-cu.

**Female genitalia** (Fig. 9D): Genitalia with 2-segmented cerci.

##### Dimensions of holotype

**(in mm).** [Measurements for the paratype, CNU-DIP-NN2013208 in brackets]. Holotype: male. NO. CNU-DIP-NN-2013215, body length 12.7 as preserved [5 as preserved]; antennae 1.5 as preserved. Forelegs: femur 3.5 as preserved; tibia 3.9. Mid leg: femur 3.9; tibia 4.2. Hind leg: femur 4.2, tibia 4.8. Wing: length 9.1 [7.4], width 3.6 [3.2]; R_2+3_ 3.5 [2.9]; bRs 2.5 [1.9]; dRs 0.35 [0.3]; R_4+5_ 4 [3.3].

##### Remarks.

The new species is similar to *Epimesoplecia shcherbakovi* Zhang, 2007, but differs from the latter in having bRs about 6–7 times as long as dRs (vs. 4.5 times); R_2+3_ clearly longer than bRs and dRs combined (vs. R_2+3_ significantly shorter than bRs and dRs combined); Rs bifurcation proximad of r-m (vs. Rs distad of r-m); M_2_ 8–11 times as long as dM_1+2_ (vs. 5 times); bM_3+4_ shorter than m-cu (vs. bM_3+4_ longer than m-cu). Comparisons with other species are listed in Table 1.

### Key to the species of *Epimesoplecia* Zhang, 2007

**Table:** 

1	Fork of Rs proximad fork of M_1+2_	**2**
–	Fork of Rs distad or at same level fork of M_1+2_	**3**
2	Fork of Rs proximad of r-m; bRs about 10 times as long as dRs	***Epimesoplecia prosoneura* sp. n.**
–	Fork of Rs distad of r-m; bRs significantly less than 10 times as long as dRs	***Epimesoplecia macrostrena* sp. n.**
3	Fork of Rs at the same level of M_1+2_; dRs shorter than r-m	**4**
–	Fork of Rs distad of M_1+2_; dRs longer than r-m	**5**
4	R_2+3_ longer than Rs; bM_1+2_ clearly shorter than m-cu	***Epimesoplecia ambloneura* sp. n.**
–	R_2+3_ distinctly shorter than Rs; bM_1+2_ longer than m-cu	***Epimesoplecia shcherbakovi* Zhang, 2007**
5	bM_1+2_ longer than dM_1+2_	***Epimesoplecia stana* sp. n.**
–	bM_1+2_ distinctly shorter than dM_1+2_	**6**
6	Antennae moniliform; bRs significantly less than 2.5 times as long as dRs	***Epimesoplecia plethora* sp. n.**
–	Antennae filiform; bRs 2.5 times as long as dRs	***Epimesoplecia elenae* Zhang, 2007**

## Discussion

**Figure 11.**
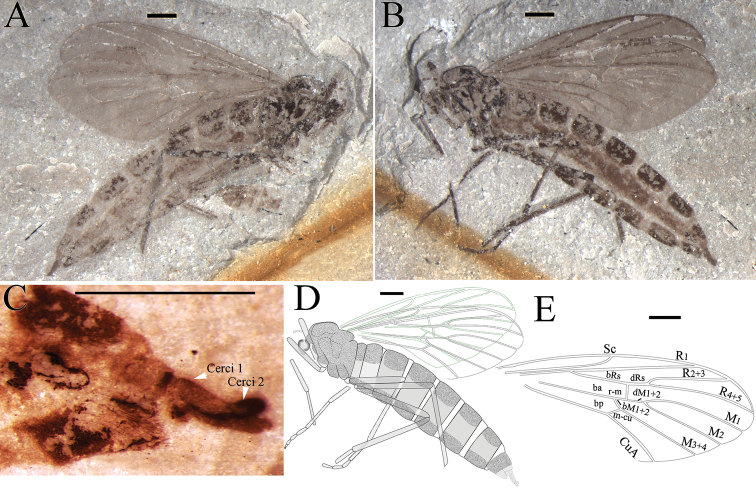
*Epimesoplecia elenae* Zhang, 2007. (CNU-DIP-NN2013213). New material. Photographs of **A, B** Part and counterpart **C** Details of female genitalia (under alcohol); Line drawings of **D** Counterpart **E** Wing. Scale bars = 1 mm.

The generic diagnosis of *Epimesoplecia* Zhang, 2007 is revised based on eleven well-preserved new specimens, among which ten are used to describe the afore-mentioned five new species. One is identified as a new material for *Epimesoplecia elenae* Zhang, 2007 (Fig. 11), In total, seven species with 13 specimens have been described in *Epimesoplecia* so far, all from the Jiulongshan Formation of China (Table 1).

It is interesting to note that only one of the 13 specimens reported so far is male, ie. the holotype of *Epimesoplecia prosoneura* sp. n. (CNU-DIP-NN2013207 p/c) with well-preserved male genitalia. We also describe a female paratype of *Epimesoplecia prosoneura* sp. n. (CNU-DIP-NN2013214) with well-preserved female genitalia. Since both specimens have similar body size, wing length and venational characters, the sexual dimorphism of this species seems to be not significant.

The measurements of body length, wing length and other important characters of wings are summarised in Table 1. The data and information suggest that the following characters are stable within a species, but differ among different species: (1) fork of Rs vs. fork of M_1+2_; (2) ratio range of bRs and dRs; (3) dRs longer or shorter than r-m; (4) bM_1+2_ longer or shorter than m-cu; and (5) antennae moniliform or filiform. Based on these taxonomic characters, a key to the species of *Epimesoplecia* Zhang, 2007 is provided.

## Supplementary Material

XML Treatment for Epimesoplecia

XML Treatment for Epimesoplecia plethora

XML Treatment for Epimesoplecia prosoneura

XML Treatment for Epimesoplecia stana

XML Treatment for Epimesoplecia macrostrena

XML Treatment for Epimesoplecia ambloneura
